# Safety and efficacy of anti-PD-L1 therapy in the woodchuck model of HBV infection

**DOI:** 10.1371/journal.pone.0190058

**Published:** 2018-02-14

**Authors:** Scott Balsitis, Volodymyr Gali, Pamela J. Mason, Susan Chaniewski, Steven M. Levine, Michael J. Wichroski, Michael Feulner, Yunling Song, Karen Granaldi, James K. Loy, Chris M. Thompson, Jacob A. Lesniak, Catherine Brockus, Narendra Kishnani, Stephan Menne, Mark I. Cockett, Renuka Iyer, Stephen W. Mason, Daniel J. Tenney

**Affiliations:** 1 Virology Discovery, Bristol-Myers Squibb, Wallingford, Connecticut, United States of America; 2 Discovery Toxicology, Bristol-Myers Squibb, Princeton, New Jersey, United States of America; 3 Discovery Toxicology, Bristol-Myers Squibb, Wallingford, Connecticut, United States of America; 4 Immunotoxicology, Drug Safety Evaluation, Bristol-Myers Squibb, New Brunswick, New Jersey, United States of America; 5 Bioanalytical Sciences, Bristol-Myers Squibb, Princeton, New Jersey, United States of America; 6 Pharmaceutical Candidate Optimization, Bristol-Myers Squibb, Princeton, New Jersey, United States of America; 7 Department of Microbiology and Immunology, Georgetown Univ. Medical Center, Washington, DC, United States of America; 8 Liver and Pancreas Tumor Center, Roswell Park Cancer Institute, Buffalo, New York, United States of America; Centre de Recherche en Cancerologie de Lyon, FRANCE

## Abstract

Immune clearance of Hepatitis B virus (HBV) is characterized by broad and robust antiviral T cell responses, while virus-specific T cells in chronic hepatitis B (CHB) are rare and exhibit immune exhaustion that includes programmed-death-1 (PD-1) expression on virus-specific T cells. Thus, an immunotherapy able to expand and activate virus-specific T cells may have therapeutic benefit for CHB patients. Like HBV-infected patients, woodchucks infected with woodchuck hepatitis virus (WHV) can have increased hepatic expression of PD-1-ligand-1 (PD-L1), increased PD-1 on CD8+ T cells, and a limited number of virus-specific T cells with substantial individual variation in these parameters. We used woodchucks infected with WHV to assess the safety and efficacy of anti-PD-L1 monoclonal antibody therapy (αPD-L1) in a variety of WHV infection states. Experimentally-infected animals lacked PD-1 or PD-L1 upregulation compared to uninfected controls, and accordingly, αPD-L1 treatment in lab-infected animals had limited antiviral effects. In contrast, animals with naturally acquired WHV infections displayed elevated PD-1 and PD-L1. In these same animals, combination therapy with αPD-L1 and entecavir (ETV) improved control of viremia and antigenemia compared to ETV treatment alone, but with efficacy restricted to a minority of animals. Pre-treatment WHV surface antigen (sAg) level was identified as a statistically significant predictor of treatment response, while PD-1 expression on peripheral CD8+ T cells, T cell production of interferon gamma (IFN-γ) upon in vitro antigen stimulation (WHV ELISPOT), and circulating levels of liver enzymes were not. To further assess the safety of this strategy, αPD-L1 was tested in acute WHV infection to model the risk of liver damage when the extent of hepatic infection and antiviral immune responses were expected to be the greatest. No significant increase in serum markers of hepatic injury was observed over those in infected, untreated control animals. These data support a positive benefit/risk assessment for blockade of the PD-1:PD-L1 pathway in CHB patients and may help to identify patient groups most likely to benefit from treatment. Furthermore, the efficacy of αPD-L1 in only a minority of animals, as observed here, suggests that additional agents may be needed to achieve a more robust and consistent response leading to full sAg loss and durable responses through anti-sAg antibody seroconversion.

## Introduction

Chronic Hepatitis B Virus (HBV) infection is a liver disease affecting approximately 250 million people globally, resulting in estimates nearing 1 million HBV-related deaths annually [1]. Chronic Hepatitis B (CHB) is associated with an inadequate immune response against the virus, including failure to achieve antibody-mediated clearance of the viral surface antigen (sAg) or robust virus antigen-specific T cell responses [2]. Multiple nucleoside/nucleotide (nuc) analogs are approved for HBV therapy [3], but while nuc therapy effectively reduces plasma HBV DNA, is reported to increase immune responses to virus [4] and reduces the incidence of serious liver disease, it does not result in appreciable clearance of infected hepatocytes [5]. Therefore, nuc therapy is lifelong and long-term efficacy can be impacted by drug resistance, side effects and viral relapse. Furthermore, patients successfully treated with nuc therapy still exhibit a higher incidence of severe liver disease than uninfected patients [6]. Additionally, severe liver inflammation can erupt upon termination of nuc therapy. In contrast, interferon-α (IFN-α) therapies can induce lifelong immunologic control of HBV in a small minority of treated patients. This ‘functional cure’ is hallmarked by clearance of circulating S antigen (HBs) and the development of anti-S antibodies (sAb). While the experience with IFN-α demonstrates that immunotherapy can achieve lasting control of HBV in CHB patients, typically less than 10% of patients achieve HBs loss within 3 years after initiating a 48 week regimen of IFN-α therapy [3,7,8], and serious side effects are associated with IFN-γ treatment [8]. Alternative immunotherapies with higher efficacy and better safety profiles are needed, and could be combined with nucs to maximize efficacy and minimize risks of immune-mediated liver damage or viral immune escape.

In contrast to the chronic infection state, immune clearance of HBV is accompanied by polyfunctional, polyspecific T cell responses against viral antigens [9–12]. The lack of robust T cell responses in CHB may be in part due to negative regulation of antiviral T cells by the T cell negative regulator PD-1, which suppresses TCR signaling upon binding to its ligands, PD-L1 and PD-L2. PD-1 expression is increased to varying degrees on virus-specific T cells in CHB [13–16]. There are varied reports of hepatic PD-L1 expression in HBV, including those reporting increases in livers of CHB patients with active hepatitis marked by serum liver enzyme elevation, but not in patients lacking evidence of liver inflammation, while upregulation of PD-L2 is not observed [17–20]. Therefore, PD-1 or PD-L1 blockade may improve T cell function in CHB patients, but the role of PD-1/PD-L1 may differ in patients with differing HBV immunity in line with their infection and immune status and treatment history. Indeed, *ex vivo* PD-L1 blockade improved responses to HBV antigens by PBMCs or intrahepatic lymphocytes from some but not all CHB patients [13,14], with effects predominantly observed in lymphocytes from patients with lower viral burdens who are reported to have greater HBV-specific T cell function [12,13]. These *in vitro* results are similar to clinical experience with PD-1 or PD-L1 blockade in cancer and Hepatitis C, where therapeutic responses affecting the course of the disease can be profound but are experienced by only a subset of subjects [21,22].

While the above results are promising, the safety and efficacy of PD-L1 blockade for CHB has not been robustly tested with relevant *in vivo* models. Preclinical *in vivo* testing of HBV immunotherapies is crucial to assess the efficacy in clearing the HBV DNA reservoir but also to assess the potential for liver damage resulting from immune cytolysis of infected hepatocytes. The infection of woodchucks with woodchuck hepatitis virus (WHV), a hepadnavirus related to HBV, has long been used as a model for pathogenesis and therapy of HBV (19). The WHV model represents the best available model for testing αPD-L1 immunotherapy as PD-1 and PD-L1 are similar to the human orthologues and their expression can be up-regulated in peripheral blood and liver of chronic WHV-infected animals [23–25]. Similar to studies of human HBV-specific T cells, *in vitro* blockade of woodchuck PD-L1 and PD-L2 during WHV antigen stimulation improved virus-specific responses in T cells from a subset of animals [25]. Additionally, αPD-L1 was tested in a woodchuck study in combination with entecavir and therapeutic DNA WHV vaccination and demonstrated antiviral effect [24]. However, interpretation of this study is difficult since it was performed with a very small numbers of animals, utilized a polyclonal αPD-L1 antisera of unknown potency, and did not robustly evaluate αPD-L1 therapy in the absence of vaccination.

To further evaluate the therapeutic potential of PD-L1 blockade in CHB, we developed a monoclonal antibody against woodchuck PD-L1 that blocks the PD-1/PD-L1 interaction. This antibody exhibits properties similar to the human monoclonal αPD-L1 antibody BMS-936559 that has demonstrated efficacy in oncology studies [26]. The anti-woodchuck PD-L1 antibody, expressed as woodchuck IgG isotype with a D265A mutation in the Fc region to ablate effector function [26], was tested in WHV-infected animals with and without entecavir co-treatment to evaluate safety and therapeutic effect. In order to assess the impact of various pre-treatment parameters on treatment response and to help identify potential biomarkers, we utilized woodchuck cohorts infected either naturally or under laboratory conditions to evaluate the effects of varying viral and immune states on response to αPD-L1.

## Methods

### Antibodies

Production and characterization of antibody wc6D5 will be described elsewhere (manuscript in preparation). Briefly, mice were immunized with purified recombinant woodchuck PD-L1 ectodomain, and hybridoma supernatants were tested for the ability to bind to woodchuck PD-L1, to block the woodchuck PD-1/PD-L1 interaction, and to recognize PD-L1 expressed on woodchuck leukocytes. Supernatants were also selected by virtue of recognizing both human and woodchuck PD-L1. This identified mouse IgG1 MAb 6D5. Antibody 6D5 was then cloned and expressed as a chimeric antibody having the same variable regions but substituting the mouse IgG1 constant regions with woodchuck IgG constant regions, with an engineered D265A mutation included to minimize effector function [27,28]. Isotype control antibody wcAnti-keyhole limpet hemocyanin (KLH) utilized the same woodchuck IgG-D265A constant regions but with variable regions targeting KLH derived from mouse IgG1 antibody KLH.1. Anti-woodchuck PD-1 antibody 27F1 (mouse IgG1) was generated by the same mouse vaccination and screening methods, and validated for flow cytometry by demonstrating staining of PMA/ionomycin-stimulated woodchuck PBMCs and blockade of staining with excess recombinant woodchuck PD-1.

### Homogeneous-time resolved fluorescence binding assays

All HTRF assays were performed at room temperature using low-volume, 384 well, non-treated, round bottom plates (Corning) in a final assay volume of 10 μL. Binding partners were diluted in HTRF assay buffer consisting of Dulbecco’s PBS (dPBS, Life Technologies) supplemented with 0.1% (w/v) Bovine Serum Albumin (Sigma) and 0.05% (v/v) Tween 20 (Bio-Rad) and 2.5 μL of each was dispensed per well. HTRF detection antibodies, LANCE^®^ Eu-W1024 Anti-Human IgG (Perkin Elmer) and SureLight^™^ Allophycocyanin-anti-6His (Perkin Elmer) were diluted in LANCE^®^ Detection Buffer and 5 μL was dispensed per well (Perkin Elmer). The final assay concentration of LANCE^®^ Eu-W1024 Anti-Human IgG and and SureLight^™^ Allophycocyanin-anti-6His were 1 nM and 20 nM, respectively. The binding assay was incubated at room temperature for 1 hour and fluorescence emission was measured at two wavelength (665 nM and 620 nM) using a Perkin Elmer EnVision 2104 Multi-Plate Reader. The FRET ratio and percent inhibition were calculated as recommended by the manufacturer’s instructions. For PD-1/PD-L1 assays, recombinant human or woodchuck PD-1 ectodomains (corresponding to amino acids 25–167 in human PD-1), each with a C-terminal human IgG epitope tag, were used at a final assay concentration of 20 nM. Recombinant human or woodchuck PD-L1 ectodomains (corresponding to amino acids 18–239 in human PD-L1), each with a C-terminal 6xHis tag, were used at a final assay concentration of 10 nM. For the CD80/PD-L1 assay, human CD80-His (Sino Biologicals) and human PD-L1-Ig (RnD Systems) were used at final assay concentrations of 80 and 10 nM, respectively. For the PD-1/PD-L2 assay, human PD-1-Ig and human PD-L2 (RnD sytems) was used at final assay concentrations of 20 and 2 nM, respectively.

### Cell binding assays

Recombinant human or woodchuck PD-1-Ig was directly labeled with R-PE using an R-PE conjugation kit (Abcam) according the manufacturer’s instructions. Human embryonic kidney cell expressing the SV40 virus large T antigen (293T) were obtained from the American Type Culture Collection. 293T clones stably expressing human PD-L1 (293T-hPDL1) or woodchuck PD-L1 (293T-wcPDL1) were seeded into PDL-coated 384 well plates (BD Falcon) at 2,000 cells per 20 μL of culture media consisting of DMEM (Life Technologies) supplemented with 10% (v/v) FBS and 1X Penicillin/Streptomycin (Life Technologies). Plates were allowed to incubate for 10 minutes at room temperature and then transferred to a 37°C/5% CO_2_ incubator for overnight incubation. The next day, antibody was added followed by the addition of 5 μL/well of PD-1-Ig-PE to cells at a final concentration of 0.5 nM. Sample were incubated for 1 hour at 37 °C for 1 hour, followed by 3 washes in 100 μl of dPBS. Cells were fixed by adding 8% (w/v) formaldehyde (Sigma), 20 μg/mL Hoechst (Thermo Scientific) in PBS for 20 minutes at room temperature. The plates were washed 3 more times in dPBS and data was obtained using a CellInsight NXT High Content Screening Platform IC903000 (Thermo Scientific). The 50% effective concentration (IC_50_) was calculated using the four-parameter logistic formula y = A+((B-A)/(1+((C/x)^D))), where A and B denote minimal and maximal % inhibition, respectively, C is the EC50, D is hill slope and x represent compound concentration.

### Animals

Woodchucks were purchased from Northeastern Wildlife (Harrison, ID) or bred at the Roswell Park Cancer Institute. Animals were housed at Northeastern Wildlife, Roswell Park Cancer Institute, or Bristol-Myers Squibb for various studies. When euthanasia was necessary, animals were euthanized by intravenous injection of Beuthanasia-D Special (Schering-Plough Animal Health).

Laboratory infected, chronic WHV+ animals were generated by subcutaneous inoculation at ~3 days of age with WHV strain 7 at a quantity of 10^7^ genome copies with subsequent monitoring of plasma anti-WHV core antibody, WHV sAg, and WHV DNA to confirm chronicity, and were used at > 1 year of age. Naturally infected animals were wild-caught and chronic infection status was confirmed by presence of anti-WHV core antibody, WHV sAg, and WHV DNA at stable levels for at least 3 months prior to use. Naive animals did not show evidence of WHV exposure, as indicated by seronegativity for anti-core and anti-sAg antibody, sAg, and WHV DNA. For acute infection studies, adult animals negative for WHV sAg, anti-WHV core antibody, and anti-WHV sAg antibody were used. Animals were inoculated intravenously with 10^7^ genome copies of WHV strain 7 virus to initiate acute infection.

### Dosing

Entecavir was dissolved at 0.1 mg/ml in a solution of 50% Phosphate Buffered Saline and 50% Woodchuck Liquid Diet (Dyets Inc, Bethlehem PA), and administered daily at 1 ml/kg via an oral dosing needle for a dose of 0.1 mg/kg/day. Antibodies were administered by slow intravenous bolus injection at a rate of ~6 ml/minute using 5 mg/ml solutions at doses as indicated in figure legends. In all studies, wc6D5 and wcAnti-KLH were administered in four doses every 3 to 4 days over a 10 day period.

### Virology

Circulating viral loads were determined by qPCR as previously described [29], or alternatively by extraction of viral DNA using the QIAamp MinElute Virus Spin Kit (Qiagen) followed by qPCR with primers WHV-SL-F: 5’-CCTCGCAGGAGAAGATCTCAA-3’, WHV-SL-R: 5’- GCAGTTGGCAGATGGAGATTG-3’, and minor groove binding probe WHV-SL-MGB: 5’-6FAM-ACCGCGTCGCAGAC-3’, within the core/polymerase overlapping region of the WHV genome. WHV sAg, anti-core antibody, and anti-sAg antibody were measured as previously described [30,31]. WHV precore eAg was measured using a commercially available HBeAg ELISA Kit (Autobio).

### Biopsies

6mm punch biopsies were collected by laparotomy from isoflurane-anesthetized woodchucks, fixed in 10% neutral buffered formalin and paraffin-embedded.

**Immunohistochemistry analysis** was performed on FFPE tissues for antibodies directed against PD-L1 (clone: SP142, Spring Bioscience, CA), CD3 (clone: LN10, Leica, IL), and MAC2 (clone: M3/38, LifeSpan, WA). The sections were manually deparaffinized through several changes of xylene and ethanol to rehydrate followed by antigen retrieval with a pressure cooker (Biocare Medical, CA) for 30 minutes at 95°C using ER1 solution (Leica, IL). The slides were then loaded on an Intellipath automatic system (Biocare Medical, CA). Endogenous peroxidase was inactivated using Peroxidazed 1 (Biocare Medical, CA) for 5 minutes. PD-L1 (1:100), CD3 (ready to use), and MAC2 (1:1000) were automatically applied to separate slides and incubated for 120, 60, and 60 minutes, respectively. The slides for PD-L1 and CD3 were then incubated with Leica Post Primary and Leica Rabbit HRP-Polymer each for 30 minutes. The slides for MAC2 were incubated with Biocare Rat HRP-Polymer (one step) for 30 minutes. Visualization was facilitated by IP FLX diaminobenzidine (DAB) chromagen. Slides were then counterstained with hematoxylin.

**Image Analysis** To quantitatively assess the expression of PD-L1, CD3, and MAC2, each entire IHC liver section from each animal was scanned at 40X magnification and digitized using Aperio ScanScope & Image Analysis System (Aperio, Vista, CA). The images were then transferred to a HALO image analysis system (Indica Lab, Corrales, NM). To quantify the expression of PD-L1, CD3, and MAC2, the entire liver section was selected, the analysis was performed by using Indica Cyto Nuclear V1.4 algorithm. Briefly, the IHC image was analyzed by color for each stain used. Nuclear stain (blue) and Positive Stain 1 (brown) were assigned to hematoxylin for total nuclear staining and DAB for the PD-L1, CD3, or MAC2 positive staining, respectively. After trained, each specific algorithm for PD-L1, CD3, and MAC2 was assigned. The percentage of Positive 1 (DAB) from each animal was used to compare among the groups.

### CBCs, plasma chemistry

Complete blood counts were analyzed on Idexx Procyte DX or Abaxis HM5 automated analyzers, or were analyzed by the Cornell University Animal Health Diagnostic Center. Alanine Aminotransferase (ALT), Aspartate Aminotransferase (AST), Alkaline Phosphatase (ALKP), Gamma Glutamyl Transferase (GGT), Total Bilirubin (TBIL), Glucose (GLUC), Urea Nitrogen (BUN), Creatinine (CREA), Total Protein (TP), Albumin (ALB), Cholesterol (CHOL), Triglycerides (TRIG), Calcium (CA), Inorganic Phosphorus (PHOS), Sodium (NA), Potassium (K), and Chloride (CL) were evaluated on an ADVIA 1800 Automated Chemistry Analyzer (Siemens Healthcare Diagnostics, Inc), or were analyzed by the Cornell University Animal Health Diagnostic Center.

### Flow cytometry

Analysis of PD-1 on total CD3+CD4-negative (CD8) T cells was performed on heparinized whole blood. 100μl of blood was stained with anti-PD-1 MAb 27F1 (3 μg/ml), followed by detection with goat anti-mouse IgG subclass 1 specific-phycoerythrin secondary antibody (Jackson ImmunoResearch). Cells were then washed and lymphocyte populations labelled with woodchuck cross-reactive anti-rat CD3-APC clone 1F4 (BD Biosciences) and anti-human CD4-PERCP clone L200 (BD biosciences), and fixation with Cal-Lyse-Whole blood Lysing Solution (Invitrogen). Flow cytometry was performed on a BD FACSVerse using FACSDiva software and analysis in FlowJo X.0.7.

### Antibody pharmacokinetics

wc6D5 in serum samples was measured by capture ELISA. Serial dilutions of serum from wc6D5-treated animals were incubated on microtiter plates coated with purified recombinant His-tagged woodchuck PD-L1, then plates were washed and wc6D5 detected with Protein G-biotin (Pierce) followed by streptavidin-HRP and detection with TMB substrate and absorbance reading at 450 nm. A standard curve of serum with known wc6D5 concentration was used to convert absorbance to antibody concentration.

### ELISPOT

PBMCs were isolated from lithium heparin-anticoagulated blood via standard Ficoll methodology. The final cell pellet was resuspended in CTS OpTimizer T-cell Expansion SFM supplemented with L-Glutamine (Life Technologies) and 10% FBS. Cells were stimulated with overlapping 15-mer peptide libraries (Genscript, Piscataway, NJ) spanning the complete coding sequence of WHV core or sAg (1μg/ml per peptide, 45 and 53 peptides per library, respectively) in 48-well plates with 2x10^6^ PBMCs in a total volume of 1ml per well and incubated at 37°C, 5% CO2 for 6 days. Following *in vitro* antigen-specific expansion, cells were resuspended by vigorous pipetting and transferred to a 2 ml 96-well block plate (Costar). Cells were collected by centrifugation at 350 x g for 10 minutes, then resuspended in 450 μl of media with 10% FBS and 15-mer peptide libraries. One-hundred and fifty microliters of cells per condition were transferred to duplicate wells of PVDF 96-well plates coated with anti-woodchuck IFN-γ monoclonal antibody 7H10 at 2 different seeding densities, 1.5e5 and 6e5 cells per well, performed in duplicate. ELISpot plates were incubated for 24 hours at 37°C, 5% CO2, then washed and spots detected with anti-IFN-γ monoclonal antibody 27C11 (subtype IgG2b, non-competitive with 7H10) followed by goat anti-mouse IgG2b specific-HRP conjugate (Jackson Immuno Research) and TMB ELISpot substrate (MabTech). Developed ELISpot plates were scanned and spot forming units quantitated using the ImmunoSpot ELISpot plate counter from CTL.

### Ethics statement

Animal work was reviewed and approved by the IACUCs at Bristol-Myers Squibb, Northeastern Wildlife and Roswell Park Cancer Center. The studies were conducted following the National Research Council’s *Guide for the Care and Use of Laboratory Animals*, the United States Department of Agriculture’s regulations CFR9 1.1–3.142, and in compliance with all state, local, and federal laws, the Animal Welfare Act, and the PHS Policy on the Humane Care and Use of Laboratory Animals, under protocol numbers 106-16WC and 1064W.

## Results

### αPD-L1 treatment in chronic WHV infection

We wished to use the chronic WHV model to evaluate the potential benefit versus risk for treatment of CHB patients with the human monoclonal antibody (HuMAb) αPD-L1, BMS-936559, previously used in oncology studies (17). However, BMS-936559 does not cross-react with woodchuck PD-L1. Therefore, we needed an antibody with reactivity to woodchuck PD-L1, as well as pharmacokinetics (PK) and effector function that would approximate the characteristics of BMS-936559 in humans. We developed a surrogate monoclonal antibody able to bind and functionally block the woodchuck PD-1/PD-L1 interaction with potency similar to that of BMS-936559 for blockade of human PD-1/PD-L1 (Table 1). This antibody, 6D5, was originally isolated as a murine IgG1 antibody that could be administered to woodchucks *in vivo* for only short durations of treatment due to the rapid induction of anti-mouse IgG antibodies. Since pharmacodynamic effects of T cell checkpoint blockade may require weeks to become evident [21,22], we generated a woodchuck-mouse chimeric variant of 6D5 (“wc6D5”), in which the antigen-binding variable domains of 6D5 were grafted onto the constant regions of woodchuck IgG. A D265A mutation in the Fc CH2 domain was included to minimize any effector function that may be present in woodchuck IgG and better model BMS-936559 activity in humans. Similar to BMS-936599, wc6D5 blocks the binding of human PD-L1 to both human PD-1 and CD80 and does not block binding of human PD-1 to PD-L2 (Table 1). The antibodies displayed comparable potency against human PD-L1 in both biochemical and cellular binding assays (Table 1). Unlike BMS-936559, wc6D5 also inhibited the woodchuck PD-1/PD-L1 interaction with potency comparable to human (Table 1). The PK of wc6D5 was evaluated in uninfected woodchucks and displayed extended PK, as expected.

**Table 1:** In vitro inhibitory activity of wc6D5.

Assay Platform	Receptor	Ligand	wc6D5 (IC_50_, nM)	BMS-936559 (IC_50_, nM)
HTRF	wcPD-1	wcPD-L1	0.46	>200
	hPD-1	hPD-L1	1.84	3.25
	hPD-1	hPD-L2	>200	>200
	hCD80	hPD-L1	1.54	1.80
Cell Binding	wcPD-1	293T-wcPD-L1	0.89	>200
	hPD-1	293T-hPD-L1	0.53	0.21

Biochemical (HTRF) and cellular binding data showing that wc6D5 inhibits both human and woodchuck PD-1 binding to PD-L1 with human PD-L1 activities comparable to BMS-936559.

**Fig 1:**
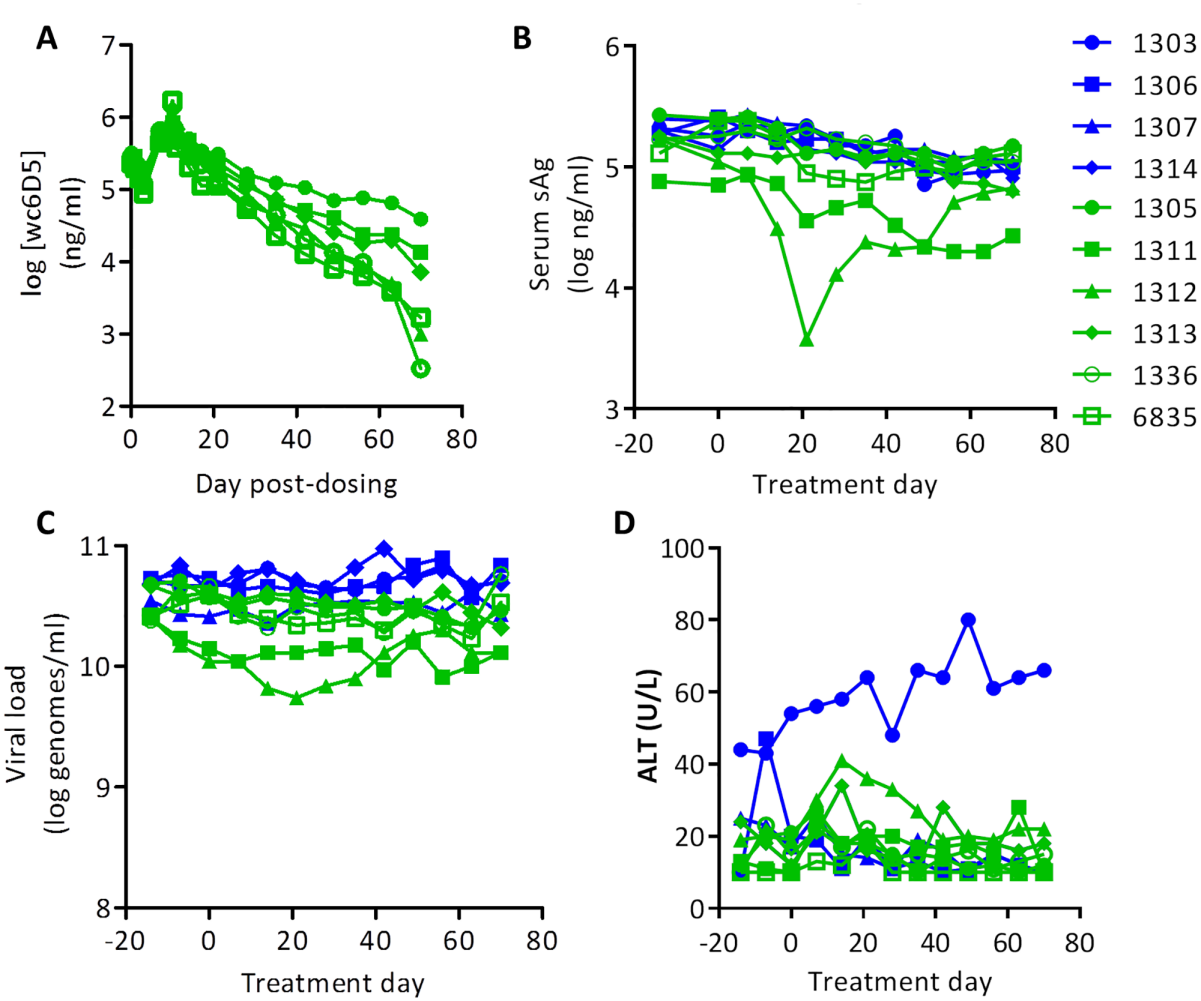
αPD-L1 monotherapy in WHV+ woodchucks. Data from control woodchucks is shown in blue, and αPD-L1-treated woodchucks in green, and symbols for each animal are the same in all panels. (A) Pharmacokinetics of antibody wc6D5 in treated woodchucks. (B) Plasma sAg levels. (C) Plasma viral load. (D) Plasma ALT. Elevated ALT in control animal 1303 was associated with increasing GGT, likely indicating tumor development in this animal.

To evaluate the effects of αPD-L1 *in vivo*, we first tested wc6D5 as a monotherapy in viremic animals with established chronic WHV infection. A cohort of 10 laboratory-bred, chronically WHV+ animals was treated with either wc6D5 (n = 6) or vehicle control (PBS, n = 4). Four doses of wc6D5 over 10 days at a 15 mg/kg/dose displayed extended PK with plasma levels exceeding the expected saturating concentration of 1 ng/mL for greater than 60 days from the start of treatment (Fig 1A). Only one of six treated animals (animal number 1312) showed a modest antiviral effect, with a transient 30-fold reduction in plasma WHV sAg accompanied by a 3-fold reduction in circulating WHV DNA, whereas viral parameters in all other animals remained unaffected. Consistent with the effect of PD-1/PD-L1 blockade in other human diseases and animal models [21,22,32,33], the antiviral effect observed in the responding animal required time to develop, with the nadir in sAg and viremia occurring at 21 days after the start of therapy (Fig 1B and 1C). This pharmacodynamic effect is in contrast to that of ETV in WHV+ woodchucks, where reductions in HBV DNA are more immediate, while reductions in sAg are less dramatic and more delayed (35). Plasma ALT was measured to determine if antiviral effect was associated with liver damage, however only a minor but notable increase in ALT was observed in the responding animal that was temporally coincident with the reduction in circulating WHV DNA and sAg (Fig 1D).

### Viral and immune states in woodchuck cohorts

**Fig 2:**
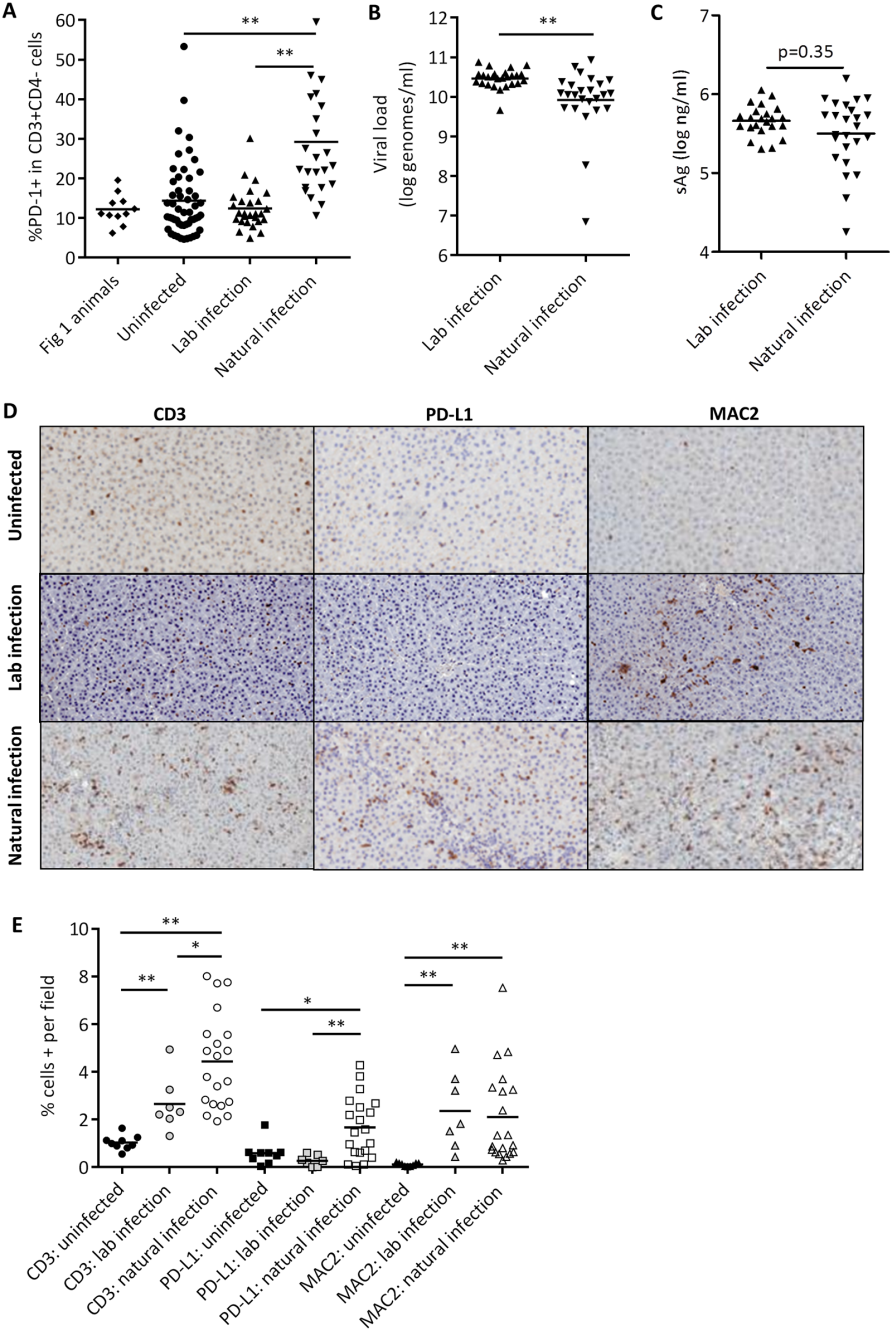
Viral and immune parameters in WHV+ cohorts. (A) pre-treatment PD-1 expression on CD8 cells (CD3+CD4-) in animals from Fig 1, compared to uninfected woodchucks (n = 52), a separate cohort of chronic WHV+ animals infected via 10^7^ genome equivalent inoculation neonatally (n = 27), or chronic WHV+ animals infected naturally (n = 24). (B) Viral load in lab-infected and naturally-infected chronic WHV+ animals. (C) sAg in lab-infected versus naturally-infected chronic WHV+ animals. (D) Representative images of liver immunohistochemistry for CD3 (left column), PD-L1 (middle column), and MAC2 (right column) from uninfected (top row), lab infected (middle row) and naturally infected (bottom row) animals. (E) Quantification of CD3, PD-L1 and MAC2 staining in 6mm liver punch biopsies from uninfected (n = 9), lab-infected (n = 7), and naturally-infected (n = 20) woodchucks. All statistical comparisons were performed with two-sided Mann-Whitney test. *, p<0.05, **, p<0.001. In A-C and E, each point represents an individual animal, with horizontal bars indicating group means in (A) and (E) geometric means in (B) and (C).

In CHB patients, PD-1 on total CD8 T cells in PBMCs and PD-L1 expression in liver are variable depending on the stage of infection, degree of inflammation, and age of the patient, suggesting that some patients, but not all, may have PD-1 dependent activity that could enable responses to PD-L1 blockade [17–20,34,35]. Thus, it is important to understand which state of human disease is best modeled by the WHV+ animals used in these studies. Previous studies reported PD-1 protein upregulation on peripheral CD3+CD4- negative (CD8) lymphocytes and PD-1 and PD-L1 RNA upregulation in the liver of WHV+ animals [23–25], but we detected no elevation of PD-1 on CD3+CD4- (CD8) lymphocytes in the laboratory-infected animals used in the experiment with results shown in Fig 1 when compared to uninfected control animals (Fig 2A). These animals all had high pre-treatment viral loads without chronic ALT or AST elevation, conditions under which PD-1 and PD-L1 are also reported to be low in humans [17,18,20,35].

We hypothesized that the neonatal infection method commonly used to generate WHV-chronic animals in laboratory settings may bias toward the establishment of high viral loads and relatively low liver inflammation, conditions in which patient T cells are least likely to respond to PD-L1 blockade *ex vivo* [14]. If so, then laboratory-infected woodchucks would not model the patients most likely to benefit from αPD-L1 therapy. In contrast, woodchucks infected naturally could offer an alternative, as they can have lower viral loads and display variable responses to adaptive immunotherapy similar to that expected for PD-L1 blockade [36]. To examine this, PD-1 expression on CD4 and CD3+CD4- (CD8) lymphocytes was compared between uninfected (n = 53), laboratory-infected (n = 27) and naturally-infected woodchucks (n = 24). PD-1 expression in CD3+CD4- cells was elevated compared to naïve controls in naturally infected woodchucks, but not in lab-infected animals (Fig 2A). PD-1 expression in CD4+ cells was similar in all animal groups (S1 Fig), resembling analyses of CHB patients which reported increased PD-1 on peripheral CD8+ T cells but not CD4+ cells [20,35], although differing patterns of PD-1 expression on CD4+ and CD8+ T cells have also been reported in humans [37]. ALT and AST levels were not elevated for any animals in either the natural or lab-infection cohorts except for animal 7254, so no pre-treatment correlations of PD-1 expression with ALT or AST could be identified. The naturally-infected cohort displayed a wider range of viral genome and sAg setpoints than the lab-infected cohort, with significantly lower circulating virus in the naturally-infected cohort (Fig 2B and 2C), albeit still with less diversity of viral setpoints than observed in human patients [38].

Expression of exhaustion markers in the liver of WHV+ animals has only been characterized at the RNA level [23], so we collected liver biopsies from uninfected (n = 9), naturally-infected (n = 20), and lab-infected (n = 7) woodchucks and characterized protein expression by immunohistochemistry for PD-L1, as well as CD3 to measure lymphocyte infiltration and MAC2 for macrophage infiltration (Fig 2D and 2E). CD3 and MAC2 were elevated in both lab- and naturally-infected animals compared to controls. However, PD-L1 was upregulated only in the naturally infected animals, and was accompanied by a greater degree of T cell infiltration. These data indicate that different WHV+ woodchuck cohorts are immunologically and virologically distinct, and only animals infected in nature display PD-1/PD-L1 upregulation. We hypothesize that it is these naturally infected animals that might best model the patients most likely to benefit from αPD-L1 treatment. Note that both the naturally-infected and naïve cohorts compared here were both wild-caught from the same source, indicating that the differences observed here are due to different modes of WHV infection and not to other environmental factors.

### Therapeutic potential of αPD-L1 in combination with ETV treatment

**Fig 3:**
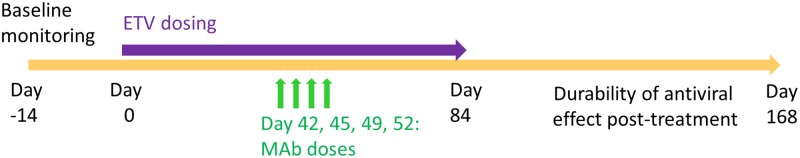
Study design for evaluation of αPD-L1 (15mg/kg) PLUS ETV (0.1 mg/kg/day) in naturally chronically-infected woodchucks.

In addition to selecting animals with PD-1/PD-L1 upregulation, co-treatment with a nucleoside analog also could improve the outcome of immunotherapy [39]. Therefore, we tested wc6D5 in a cohort of naturally-infected woodchucks that were treated with ETV in combination with either wc6D5 (n = 11), or isotype control antibody wcAnti-KLH (n = 5). The study design is shown in Fig 3. ETV was administered via daily oral dosing at 0.1 mg/kg for 12 weeks, with wc6D5 therapy starting after 6 weeks of ETV treatment. An additional 10 weeks of post-ETV treatment monitoring was included in the study to examine duration of effect after cessation of nucleoside analog antiviral therapy. Animal 7254 had 1–2 logs lower baseline viral load than other animals, so ETV treatment was not administered to this animal during the first four weeks of the ETV treatment phase to avoid driving viral load to undetectable levels with ETV alone [40]; treatments were otherwise synchronous with the remainder of the cohort. Plasma WHV DNA and sAg were monitored throughout and levels of WHV eAg were evaluated at selected time points during the study.

**Fig 4:**
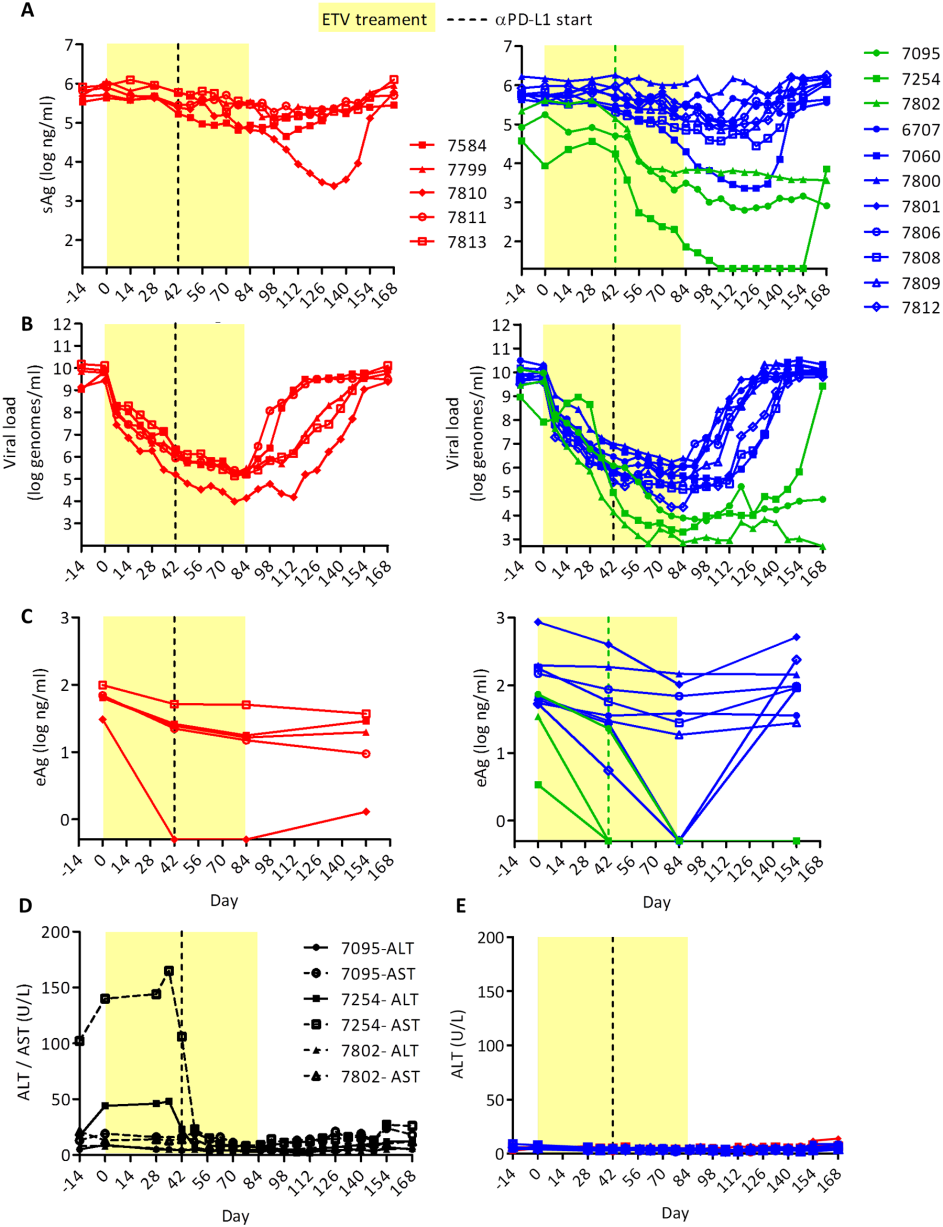
Antiviral effects of αPD-L1 in naturally infected, ETV-treated woodchucks. (A) Plasma sAg, (B) viral loads, and (C) eAg in animals receiving ETV plus isotype control antibody (left panel A-C) and ETV plus αPD-L1 antibody (right panel A-C). Controls are shown in red. αPD-L1 treatment responders are in green; αPD-L1 non-responders (define) are in blue. Horizontal axes indicate limits of detection in all plots: 500 copies/ml for viral load, 20 ng/ml for sAg, and 0.5 ng/ml for eAg. (D) Plasma AST and ALT values in treatment-responding animals 7095, 7254, and 7802. (E) ALT values in control animals and non-responder animals treated with αPD-L1.

As expected, wc6D5 displayed extended pharmacokinetics with detectable antibody in serum (>200 ng/mL) for 5–12 weeks in treated animals (S2 Fig). ETV plus isotype control antibody treatment had minimal effect on WHV sAg levels but reduced plasma WHV DNA by > 4 logs. In contrast, three animals (#7095, 7254, and 7802) in the ETV plus wc6D5 group showed apparent antiviral responses characterized by sharp declines in sAg within 3 weeks of the start of wc6D5 therapy and prolonged post-treatment suppression of viral parameters (Fig 4A and S2 Fig). This pattern of effects was not observed in any ETV treated only control animal. Animals were classified as responders to αPD-L1 if (1) sAg declines were temporally associated with the start of PD-L1 treatment, (2) rebound of sAg, eAg, and viral load either did not occur (7254 and 7802, 7095 for eAg) or were delayed compared to the control animals (7095 for vDNA and sAg). Antiviral effects in αPD-L1 responders were sustained for many weeks, with 2 of 3 responding animals maintaining low plasma WHV DNA and sAg levels until the end of the study. Although anti-sAg antibodies did not appear in any of the treated animals, plasma WHV DNA and sAg in the responder animals either did not rebound after stopping ETV therapy (#7095 and 7802), or rebounded 50–60 days after cessation of ETV (#7254), much later than controls and non-responding animals in the wc6D5 treatment group (Fig 4B). eAg levels declined to below the limit of detection during ETV therapy in one control animal and several animals in the wc6D5 group. However, eAg remained undetectable after ETV withdrawal only in the animals with sAg and viral DNA responses to wc6D5, while eAg rebounded in all other animals (Fig 4C).

A major concern with any novel immunotherapy for CHB is that induced immune responses could cause unacceptable liver damage in the process of controlling viral infection. However, no marked increase in ALT or AST levels occurred in the three treatment-responding woodchucks during combined ETV plus wc6D5 therapy (Fig 4D), or in any of the other ETV plus wc6D5-treated animals. Additionally, no overt clinical illness or signs of toxicity in complete blood counts or plasma chemistry profiles were observed in any of the ETV plus wc6D5-treated animals. These results suggest that therapeutic responses in viral and immune markers can occur in the absence of unacceptable hepatic damage.

### Biomarkers of response to αPD-L1 treatment

**Fig 5:**
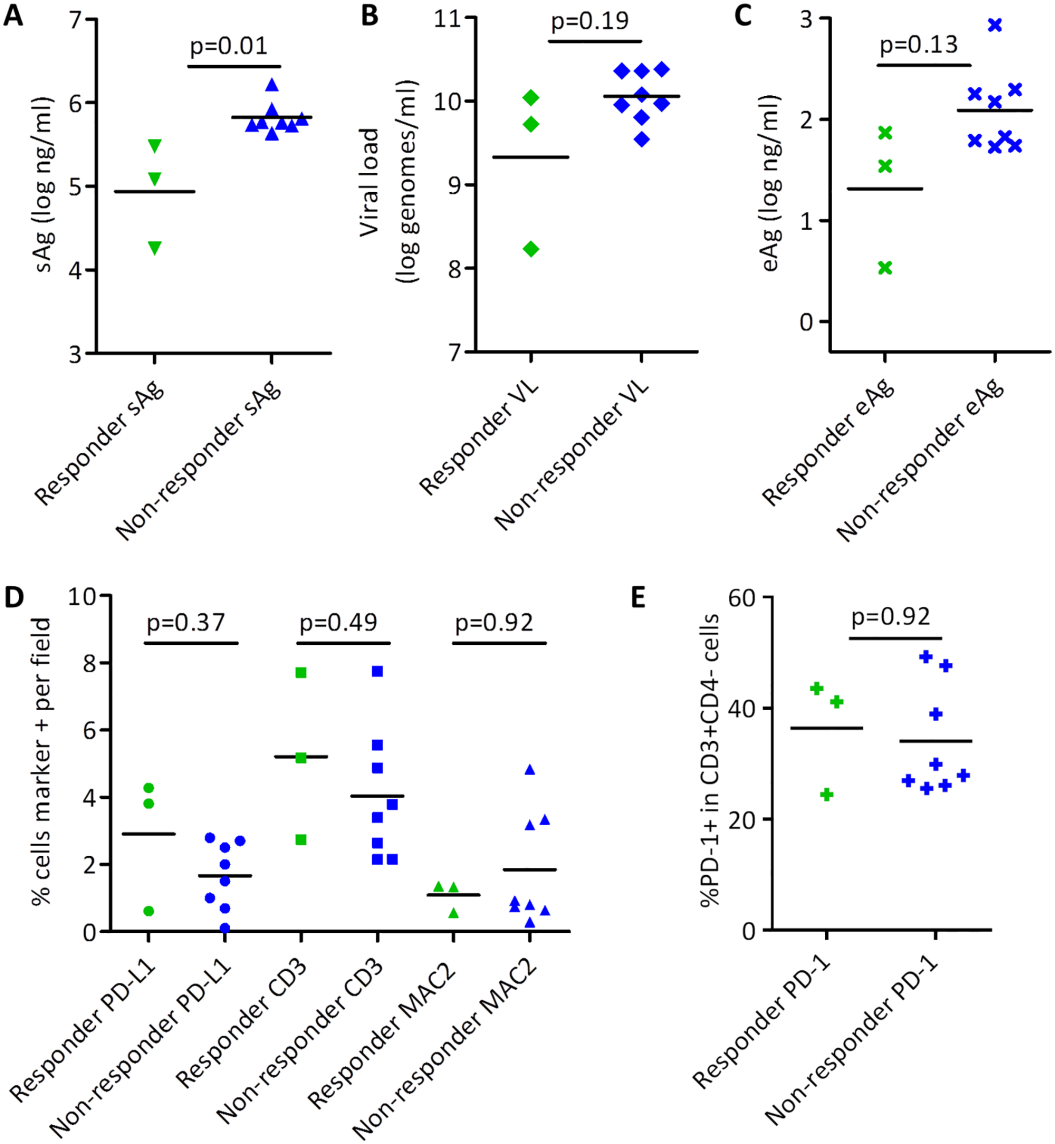
Biomarkers of response. Pre-treatment sAg (A), viral load (B), eAg (C), liver IHC markers PD-L1, CD3, and MAC2 (D), and PD-1 on peripheral CD3+CD4- (CD8) T cells (E) were compared between αPD-L1-treated animals that showed a treatment response (green) and those that did not (blue). Statistical comparisons were made using two-sided Mann-Whitney test.

While αPD-L1 is a promising therapy for chronic infections, significant immune-related side effects can occur [21,22]. Identification of biomarkers that identify patients most likely to respond to treatment could maximize the benefit/risk involved in αPD-L1 therapy. We compared pre-treatment plasma sAg, viral load, eAg, PD-1 on peripheral CD3+CD4- (CD8) cells, and liver immunohistochemistry for PD-L1, CD3, and MAC2 between treatment-responsive and non-responsive animals in the wc6D5 plus ETV treatment group (Fig 5). Activity of PBMCs in IFN-γ ELISPOT assays were also assessed [S3, S4, S5, S6, and S7 Figs]. Of these parameters, only pre-treatment sAg showed a statistically significant difference between these two groups (Fig 5A). Plasma eAg and liver PD-L1 were not statistically significant differentiators between the two groups in this analysis but may nevertheless merit additional investigation, as both of these markers had two of the three responder animals showing lower and higher values, respectively, than all other animals (Fig 5C and 5D). However, the small number of treatment-responsive animals limits the statistical power of these comparisons.

Notably, PD-1 on peripheral CD8 T cells, ELISPOT responses of PBMCs to WHV peptide pools, and duration of detectable wc6D5 exposure had no apparent association with *in vivo* treatment response (Fig 5E, S2, S3, S4, S5, S6, and S7 Figs). Since WHV-specific MHC-peptide (e.g., tetramer) reagents are not available, PD-1 on total CD8 T cells does not identify virus-specific versus non-specific T cells, and thus may serve more as a marker of general peripheral inflammation than of virus-specific immune exhaustion or abundance of virus-specific T cells. Although ELISPOT assays performed on PBMC throughout the study showed a range of responses, no discernable pattern emerged to distinguish wc6D5 responders, wc6D5-treated non-responders, and controls (S3, S4, S5, S6, and S7 Figs). The lack of correlation between activity in peripheral ELISPOTs and *in vivo* efficacy could reflect differences in the peripheral versus liver immune environments, or could result from inter-assay variability in the ELISPOT assay masking weak virus-specific signals.

### Safety of αPD-L1 treatment during acute WHV infection

**Fig 6:**
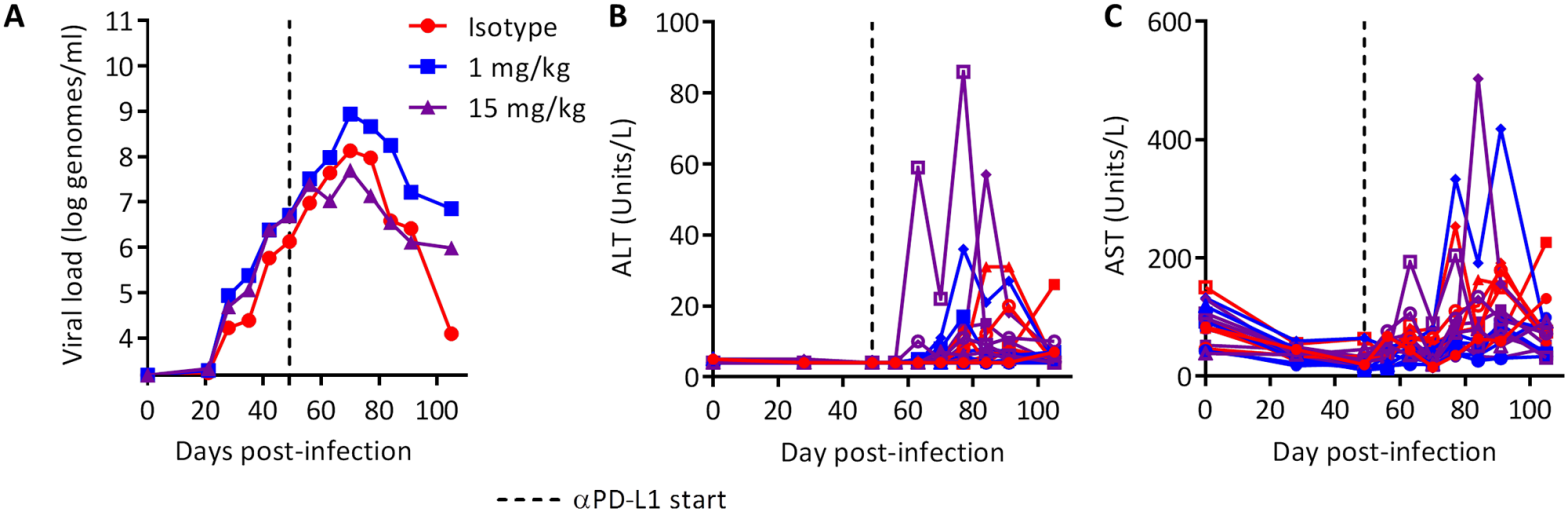
Safety of αPD-L1 in acute WHV infection. (A) Group geometric mean viral loads in acutely infected woodchucks treated with αPD-L1 at 1 or 15 mg/kg on days 49, 52, 56, and 59. No statistically significant differences were noted. (B) ALT and (C) AST values in acutely infected woodchucks with and without anti-PD-L1. Colors match the groups shown in (A).

To further evaluate the potential for liver damage following PD-L1 blockade, we evaluated the effects of wc6D5 on animals with acute WHV infection, when immune responses are much more robust than in chronic infection and the possibility of disease due to hyper-immune activity is therefore greater. Twenty adult, WHV-naïve animals were inoculated intravenously with 10^7^ genome equivalents of WHV7 and plasma WHV DNA, circulating levels of liver enzymes, and clinical condition were monitored. Antibody treatment was initiated 7 weeks post-infection, approximately 3 weeks before peak viral load and the onset of immune clearance (Fig 6A, S8 Fig). Seven animals were treated with wc6D5 at 15 mg/kg/dose, seven received wc6D5 at 1 mg/kg/dose, and six control animals received wcAnti-KLH at 15 mg/kg/dose. Serum exposure of wc6D5 was much greater with the 15 mg/kg dose than at 1 mg/kg, and the half-life was much shorter with 1 mg/kg (S9 Fig) S9 Fig. No significant differences were observed in plasma WHV DNA between groups. However, large inherent variability in the kinetics of acute WHV infection interfere with such an analysis [24], and effects of αPD-L1 on viral kinetics in individual animals cannot be ruled out (S8 Fig). Plasma levels of liver enzymes AST and ALT increased in some animals in all three treatment groups during immune clearance of WHV, although to levels generally lower than those observed during acute HBV infection in humans. Peak AST or ALT values were higher in four out of fourteen wc6D5-treated animals than the peaks observed in the control animals, but only by 2- to 3-fold (Fig 6B and 6C). The small magnitude of this difference makes it unclear if this represents an actual pharmacodynamic consequence of PD-L1 blockade or just natural variability in AST and ALT peaks during clearance of acute infection. Combined with data from the chronic WHV studies, these data suggest that αPD-L1 treatment is likely to improve viral control without large AST and ALT increases.

## Discussion

Novel immunotherapies have the potential to improve upon the standard of care for CHB patients. Interferon therapy shows that immunomodulation can achieve long-term viral control with a limited treatment duration, something that nucleoside analog therapy rarely achieves [3]. However, improvements in the efficacy and tolerability of immunotherapeutic approaches are needed for more widespread use.

PD-L1 blockade has been tested in the HBV DNA hydrodynamic injection model in mice. Virus clearance was accelerated when treatment was initiated prior to DNA injection; treatment after establishment of chronicity was not tested [41]. Only one previous publication evaluated *in vivo* efficacy of αPD-L1 in woodchucks [24]. In that study, monotherapy with αPD-L1 in three WHV+ woodchucks reportedly showed no efficacy. This is similar to our observation of only a modest and transient effect in 1 of 6 animals receiving αPD-L1 monotherapy. An antiviral effect was observed when αPD-L1 was combined in a triple therapy with therapeutic vaccination and ETV treatment, but the dual combination of ETV and αPD-L1 without vaccination was not tested. Therefore it is unclear if therapeutic vaccination was necessary for efficacy. Additionally, a polyclonal rabbit αPD-L1 antiserum was used to block PD-L1 in these studies and it is unclear if the antisera was able to fully block PD-1/PD-L1 interactions *in vivo* or if antibodies against rabbit IgG produced by the recipient woodchucks (anti-drug antibodies) may have limited the duration of PD-L1 blockade and therapeutic effect. Lastly, like many studies with WHV+ woodchucks, only a very small numbers of animals were included in each treatment group, so an infrequent response in a subset of animals may not necessarily have been detected. We expand upon this earlier work by using a well-characterized monoclonal antibody to achieve robust and prolonged PD-L1 blockade, having a sufficient number of animals to detect responses in a minority of treated animals, and characterizing multiple peripheral and hepatic parameters in each study as well as WHV-naïve, lab-infected and naturally-infected WHV+ animals.

Sustained antiviral effects were seen with αPD-L1 plus ETV treatment that were not observed with ETV treatment alone. Large reductions in plasma sAg occurred shortly after initiation of PD-L1 blockade in 3 of 11 treated animals. This effect was maintained after cessation of ETV treatment in these same animals. Similar reductions and/or continued suppression also was observed for viral DNA and eAg. In 2 of the responder animals (#7095 and 7802), these benefits continued well after wc6D5 was cleared from circulation (compare Fig 4, S2 Fig), indicating that PD-L1 blockade can induce durable antiviral effects even after withdrawal of therapy. In contrast, the third responder (#7254) experienced rebound of both sAg and circulating WHV DNA shortly after plasma wc6D5 fell below detectable levels (S3 Fig). The antiviral effects of αPD-L1 demonstrated here are similar to those observed in responders to IFN-γ treatment in CHB patients, where eAg loss and improved control of viremia occur in approximately 20–40% of treated patients with or without nucleoside analog antiviral co-treatment [3,7,39]. Altogether, our results demonstrate that αPD-L1 has a previously unrecognized therapeutic benefit in WHV+ animals that is achieved without combination with other immunotherapies. In contrast to the efficacy with ETV plus αPD-L1 in naturally-infected animals, little efficacy was observed when lab-infected animals were treated with αPD-L1 monotherapy. We did not have sufficient animals available to test ETV plus αPD-L1 in lab-infected animals, so we were not able to formally demonstrate whether the difference in efficacy was due to the lack of ETV co-treatment, the virologic and immunologic differences between woodchuck cohorts, or both. On one hand, it is unsurprising that αPD-L1 would have limited efficacy in animal cohorts that do not display increased hepatic PD-L1 protein expression. However, ETV treatment was recently shown to be synergistic with TLR9 agonist therapy in chronically-infected woodchucks [42], supporting the possibility that nucleoside analog antiviral co-treatment may bolster the benefits of a range of immunotherapies.

The response rate observed here with αPD-L1 plus ETV (3/11 animals, 27%) is similar to the response rate observed in a phase 1 clinical trial of αPD-1 (nivolumab) in HCV-infected subjects (3/20 at 10 mg/kg dose) [22], the rate of response to PD-1/PD-L1 blockade as monotherapy in oncology studies [21,33,43,44], and the *ex vivo* rate of response when αPD-L1 is applied to woodchuck PBMCs *ex vivo* [25]. In the oncology setting, accumulating evidence indicates that the response to PD-1 or PD-L1 blockade is limited by both the abundance of tumor-reactive T cells whose function can be potentiated by treatment [45–47], and the involvement of multiple negative T-cell regulatory pathways [48,49]. Similar considerations may apply to CHB, where the abundance of virus-reactive T cells is variable [12–14], and other T-cell regulatory pathways have been reported to regulate virus-specific T cells [15,16,37].

In humans, upregulation of PD-1 or PD-L1 is not observed in all CHB patients, but rather correlates with inflammation and immune-active disease [17–20,35]. Differences in immune and viral parameters between WHV+ cohorts suggest that lab-infected and naturally-infected woodchucks may model different CHB patient subgroups. In CHB patients, PD-1 on total CD8 T cells in PBMCs and PD-L1 expression in liver are variable depending on the stage of infection and age of the patient, suggesting that some, but not all patients may have PD-1/PD-L1 pathway dependent activity required for responses to PD-L1 blockade [15,17–20,34,35]. In particular, CHB patients with the highest circulating virus are least likely to respond to PD-L1 blockade [14]. Elevated expression of PD-1 on CD3+CD4- (CD8) T cells during WHV infection was previously reported [24,25] and was also observed in our studies, albeit not in all animal cohorts. Other groups have reported conflicting data on whether PD-L1 RNA is upregulated in the livers of WHV+ animals [23,25], which may be explained by variability among woodchuck cohorts similar to that observed here. Our results also closely parallel a study where naturally-infected WHV+ woodchucks were used to test the effect of hepatocyte-expressed IL-12, and a correlation between baseline virologic state and immunotherapeutic response was observed [36]. Furthermore, reduction in circulating virus during nuc therapy is also accompanied by enhanced antiviral immunity in WHV [50] and HBV [51]. Although the reasons why natural and lab infection result in different immune states in woodchucks are unclear, potential factors include the infecting strain of WHV, differences in route of inoculation (s.c. inoculation in the lab versus exposure most likely during birth), and the inoculum dose. The data presented here demonstrate the value of assessing a range of viral and immune states when testing immunotherapies that potentiate the effects of pre-existing T cells.

Although the reductions in WHV DNA and WHsAg reported here with αPD-L1 therapy are encouraging, they stop short of the ultimate goal of anti-sAg antibody seroconversion. No induction of anti-sAg antibody occurred in any of the chronically infected woodchucks treated with wc6D5, although the study did not have sufficient numbers of animals to detect seroconversion rates similar to those seen with IFN treatment (~10%). Perhaps the study was terminated prior to achieving levels of WHsAg and CCC DNA reduction necessary for seroconversion. Predictably, greater efficacy may also be achieved by using αPD-L1 in combination with other novel therapies including therapeutic vaccines and antivirals [24] or antigen reducing strategies that may also synergize with immunotherapy [52].

Meanwhile, it may be possible to identify patient subgroups in which αPD-L1 achieves larger or more consistent therapeutic effects. Low pre-treatment viremia (below 10^6^ copies/mL) is a predictor of *ex vivo* response to αPD-L1 with human intrahepatic lymphocytes [14]. Plasma viral load and eAg level were not statistically significant predictors of response in the woodchuck studies presented here. This might be a consequence of low statistical power due to limited animal wability rather than a true lack of association. However, a lower pre-treatment level of the viral sAg was a statistically significant predictor of treatment response. In contrast, no measured immune parameter was associated with treatment response, despite evaluation of both peripheral T cell function via ELISPOT and examination of liver immune status by immunohistochemistry. Differences between peripheral and intrahepatic immune states, the multifactorial nature of T cell regulation, and the limited sample sizes used may have limited our ability to identify a single predictive immune parameter. However, it is likely that the viral parameters represent a surrogate readout of immune state, wherein lower viral load and sAg antigenemia result from greater underlying antiviral immune responses, and thus correlate with response to αPD-L1 therapy. Using these readily accessible biomarkers to identify patients likely to benefit from αPD-L1 treatment is a promising avenue for further investigation.

A large percentage of the total hepatocytes in the liver can be infected by HBV [53] or WHV [40]. Hence, a primary concern for treatment of CHB with a novel immunotherapy is that the activation of cytotoxic clearance of infected cells might result in severe liver damage. Alternatively, generalized immune activation could lead to other dose-limiting effects, as was recently reported in woodchucks treated with a TLR7 agonist [31]. Importantly, the beneficial effects of αPD-L1 observed here occurred without any overt signs of hepatotoxicity. When wc6D5 was administered as a monotherapy, minor ALT and AST increases were observed in the one animal that had a transient antiviral response. In contrast, αPD-L1 plus ETV achieved greater therapeutic effect without detectable elevation in AST or ALT or any other evidence of side effects. Even in the context of acute WHV infection, when antiviral T cell responses are expected to be the most robust, αPD-L1-treated animals did not develop serious liver disease. This positive safety profile is encouraging for the use of αPD-L1 as a building block of highly effective combination therapies.

In summary, treatment with αPD-L1 demonstrated a positive balance of efficacy and tolerability in this study. Further pre-clinical and clinical studies are warranted to identify patient groups that will achieve the greatest benefit from PD-1/PD-L1 blockade, and to test potential combinations with other experimental therapies.

## Supporting information

S1 Fig Pre-treatment PD-1 expression on CD3+CD4+ cells in uninfected woodchucks, chronic WHV+ animals infected via 10^7^ genome equivalent inoculation neonatally, or chronic WHV+ animals infected naturally. (TIF)

S2 Fig Pharmacokinetics and pharmacodynamics in animals receiving ETV + αPD-L1 therapy. (A) Plasma levels of anti-woodchuck PD-L1 Mab wc6D5 was determined in plasma of treated animals at various times post-infection. Treatment responders are shown in green; animals treated with wc6D5 that did not show an antiviral response are in blue. (B-D) Comparison of wc6D5 PK with viral load and sAg kinetics in treatment responders. Individual graphs are shown for each animal. sAg data are shown at left, viral load data are shown at right. Horizontal axes are at the limit of detection for each parameter: 200 ng/ml for wc6D5, 500 copies/ml for viral load, and 20 ng/ml for sAg.
(TIF)

S3 Fig ELISPOT results in animals treated with ETV + aPD-L1. PBMCs were isolated and tested for responses to WHV core and sAg peptide libraries as described in Materials and Methods. Results for each individual woodchuck are shown. ELISPOT responses are displayed as fold change over unstimulated controls for WHV cAg library (blue bars) and sAg library (red bars). Viral load (green) and sAg (yellow) in the same animal is overlaid on each graph. wc6D5 treatment responder animals 7095, 7254, and 7802.
(TIF)

S4 Fig ELISPOT results in animals treated with ETV + aPD-L1. PBMCs were isolated and tested for responses to WHV core and sAg peptide libraries as described in Materials and Methods. Results for each individual woodchuck are shown. ELISPOT responses are displayed as fold change over unstimulated controls for WHV cAg library (blue bars) and sAg library (red bars). Viral load (green) and sAg (yellow) in the same animal is overlaid on each graph. wc6D5 treatment non-responder animals 6707, 7060, 7800, and 7802.
(TIF)

S5 Fig ELISPOT results in animals treated with ETV + aPD-L1. PBMCs were isolated and tested for responses to WHV core and sAg peptide libraries as described in Materials and Methods. Results for each individual woodchuck are shown. ELISPOT responses are displayed as fold change over unstimulated controls for WHV cAg library (blue bars) and sAg library (red bars). Viral load (green) and sAg (yellow) in the same animal is overlaid on each graph. Antibody wc6D5 treatment non-responder animals 7806, 7808, 7809, 7801.
(TIF)

S6 Fig ELISPOT results in animals treated with ETV + aPD-L1. PBMCs were isolated and tested for responses to WHV core and sAg peptide libraries as described in Materials and Methods. Results for each individual woodchuck are shown. ELISPOT responses are displayed as fold change over unstimulated controls for WHV cAg library (blue bars) and sAg library (red bars). Viral load (green) and sAg (yellow) in the same animal is overlaid on each graph. Antibody isotype-control treated animals 7799, 7811, and 7813.
(TIF)

S7 Fig ELISPOT results in animals treated with ETV + aPD-L1. PBMCs were isolated and tested for responses to WHV core and sAg peptide libraries as described in Materials and Methods. Results for each individual woodchuck are shown. ELISPOT responses are displayed as fold change over unstimulated controls for WHV cAg library (blue bars) and sAg library (red bars). Viral load (green) and sAg (yellow) in the same animal is overlaid on each graph. Antibody isotype-control treated animals 7584 and 7810.
(TIF)

S8 Fig Individual viral loads in acutely infected woodchucks. Animals undergoing acute WHV infection were treated with wc6D5 at 1 mg/kg (blue) or 15 mg/kg (purple), or with isotype control MAb wc6D5 at 15 mg/kg (red). Antibodies were administered in four doses over 10 days, starting on week 7 post-WHV infection. Viral loads for each individual animal in each group are shown.
(TIF)

S9 Fig Pharmacokinetics of antibody wc6D5 in acutely infected woodchucks. Plasma levels of anti-woodchuck PD-L1 mAb wc6D5 was determined in plasma of treated animals at various times post-infection. Animals received either 1 mg/kg (blue) or 15 mg/kg (purple) wc6D5 in four doses between days 49 and 59 post infection.
(TIF)
