# Supplementary figures and images for: Safety and efficacy of anti-PD-L1 therapy in the woodchuck model of HBV infection
Source: PLoS One. 2018 Feb 14;13(2):e0190058. doi: 10.1371/journal.pone.0190058 (PMC5812555; doi:10.1371/journal.pone.0190058)

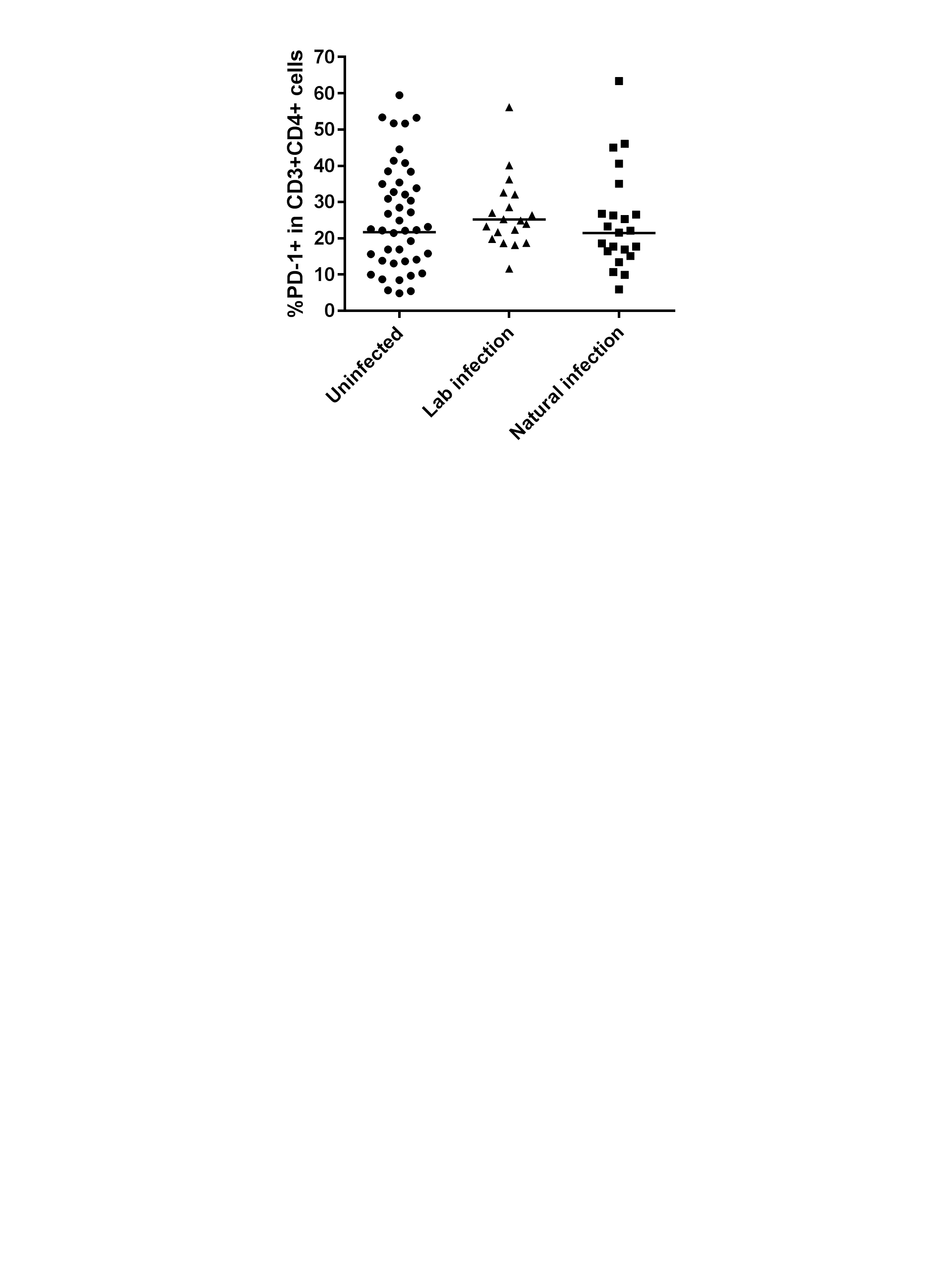

Supplement: S1 Fig — (TIF) [file pone.0190058.s001.TIF]

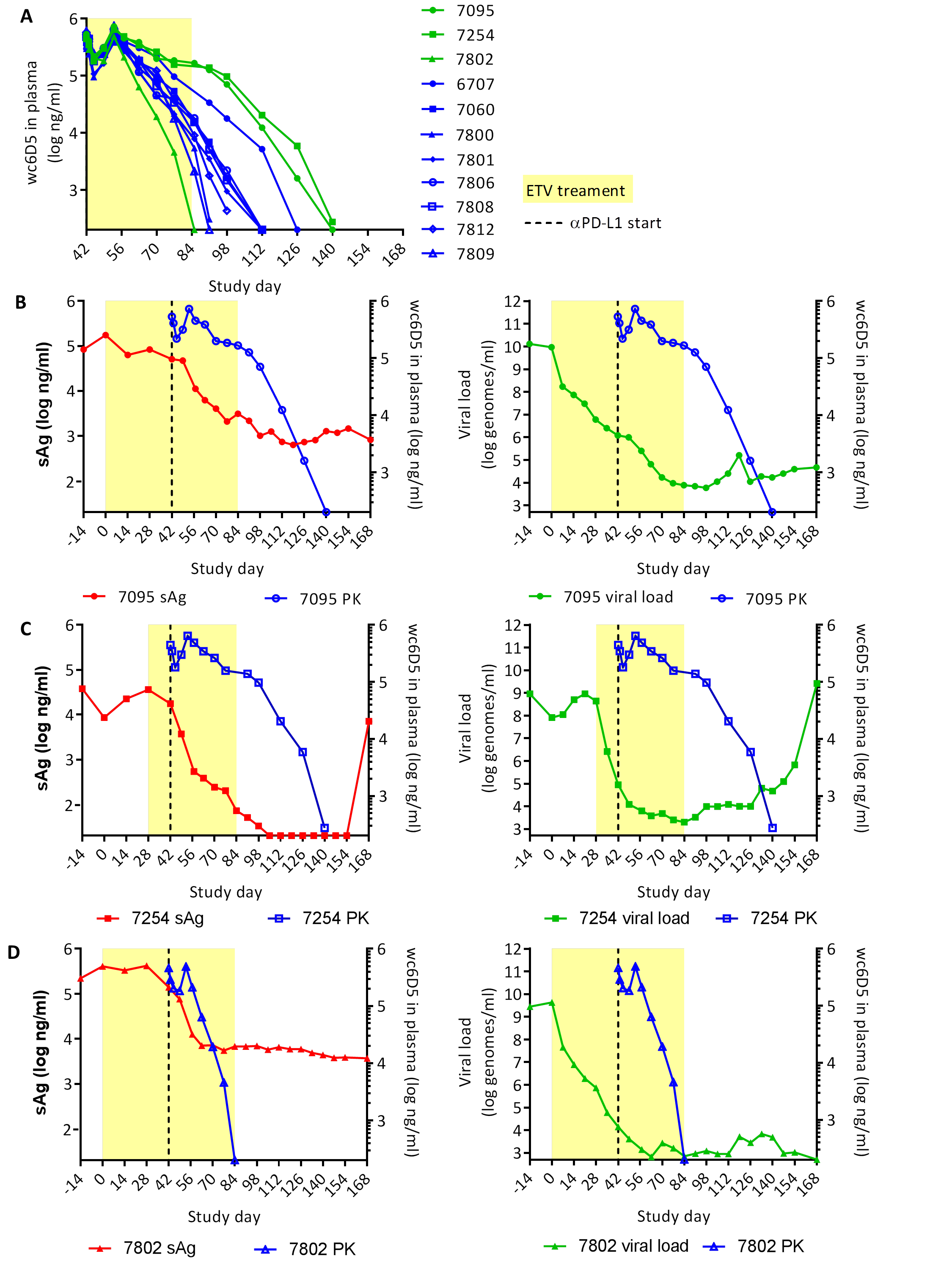

Supplement: S2 Fig — (A) Plasma levels of anti-woodchuck PD-L1 Mab wc6D5 was determined in plasma of treated animals at various times post-infection. Treatment responders are shown in green; animals treated with wc6D5 that did not show an antiviral response are in blue. (B-D) Comparison of wc6D5 PK with viral load and sAg kinetics in treatment responders. Individual graphs are shown for each animal. sAg data are shown at left, viral load data are shown at right. Horizontal axes are at the limit of detection for each parameter: 200 ng/ml for wc6D5, 500 copies/ml for viral load, and 20 ng/ml for sAg. (TIF) [file pone.0190058.s002.TIF]

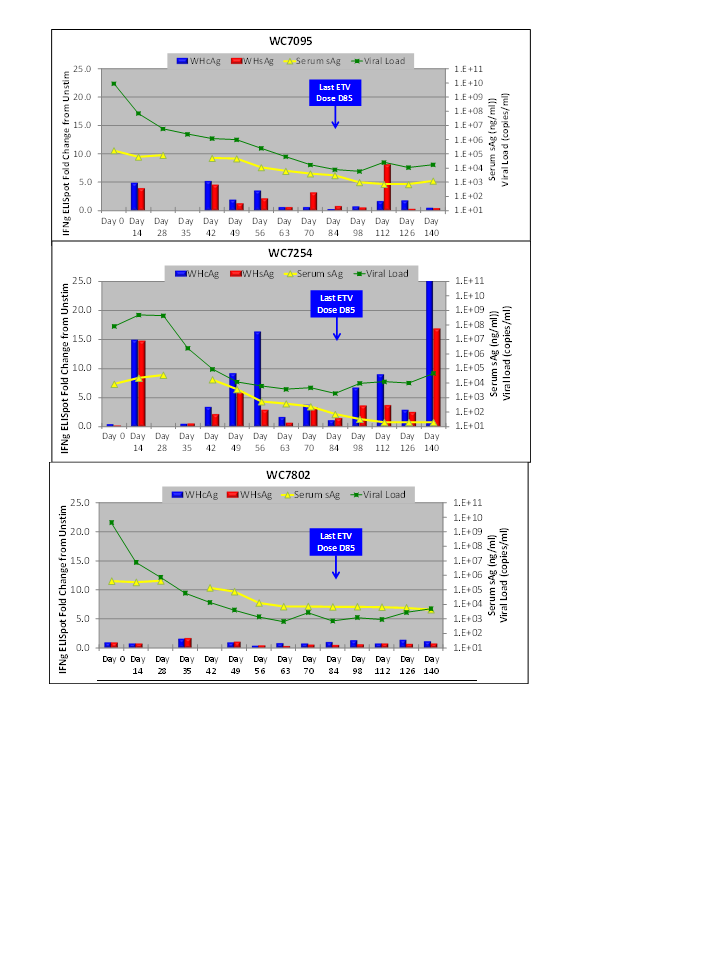

Supplement: S3 Fig — PBMCs were isolated and tested for responses to WHV core and sAg peptide libraries as described in Materials and Methods. Results for each individual woodchuck are shown. ELISPOT responses are displayed as fold change over unstimulated controls for WHV cAg library (blue bars) and sAg library (red bars). Viral load (green) and sAg (yellow) in the same animal is overlaid on each graph. wc6D5 treatment responder animals 7095, 7254, and 7802. (TIF) [file pone.0190058.s003.tif]

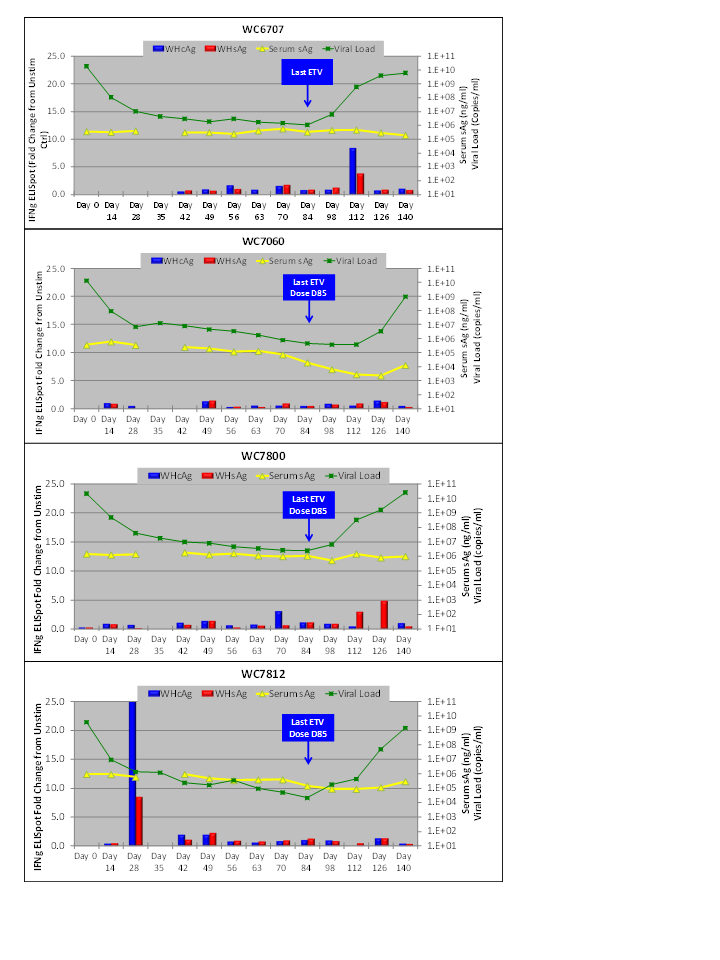

Supplement: S4 Fig — PBMCs were isolated and tested for responses to WHV core and sAg peptide libraries as described in Materials and Methods. Results for each individual woodchuck are shown. ELISPOT responses are displayed as fold change over unstimulated controls for WHV cAg library (blue bars) and sAg library (red bars). Viral load (green) and sAg (yellow) in the same animal is overlaid on each graph. wc6D5 treatment non-responder animals 6707, 7060, 7800, and 7802. (TIF) [file pone.0190058.s004.tif]

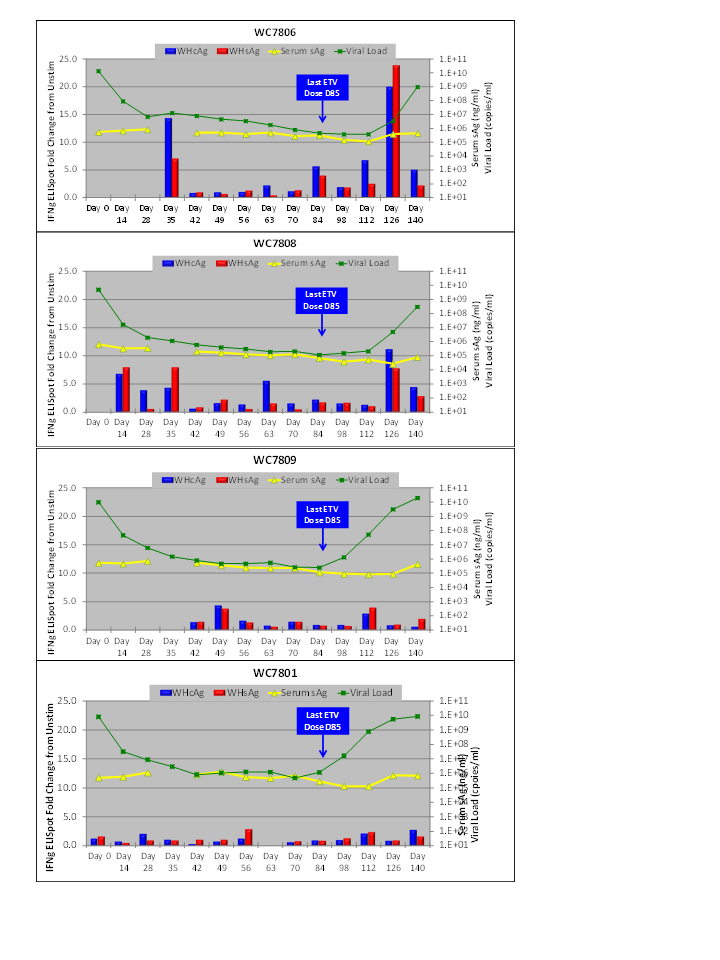

Supplement: S5 Fig — PBMCs were isolated and tested for responses to WHV core and sAg peptide libraries as described in Materials and Methods. Results for each individual woodchuck are shown. ELISPOT responses are displayed as fold change over unstimulated controls for WHV cAg library (blue bars) and sAg library (red bars). Viral load (green) and sAg (yellow) in the same animal is overlaid on each graph. Antibody wc6D5 treatment non-responder animals 7806, 7808, 7809, 7801. (TIF) [file pone.0190058.s005.tif]

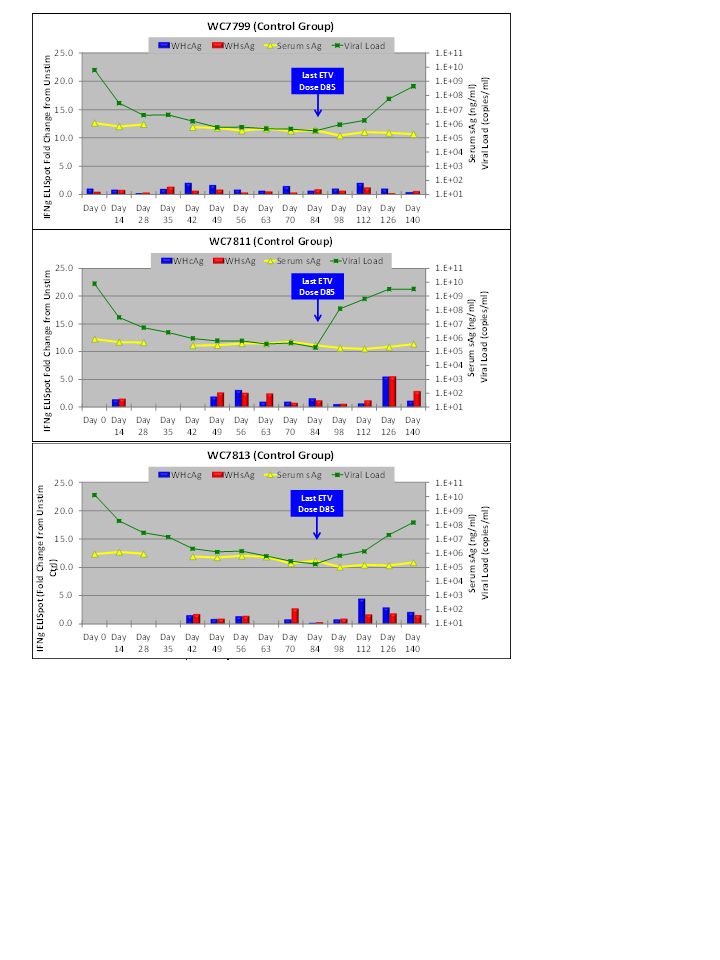

Supplement: S6 Fig — PBMCs were isolated and tested for responses to WHV core and sAg peptide libraries as described in Materials and Methods. Results for each individual woodchuck are shown. ELISPOT responses are displayed as fold change over unstimulated controls for WHV cAg library (blue bars) and sAg library (red bars). Viral load (green) and sAg (yellow) in the same animal is overlaid on each graph. Antibody isotype-control treated animals 7799, 7811, and 7813. (TIF) [file pone.0190058.s006.tif]

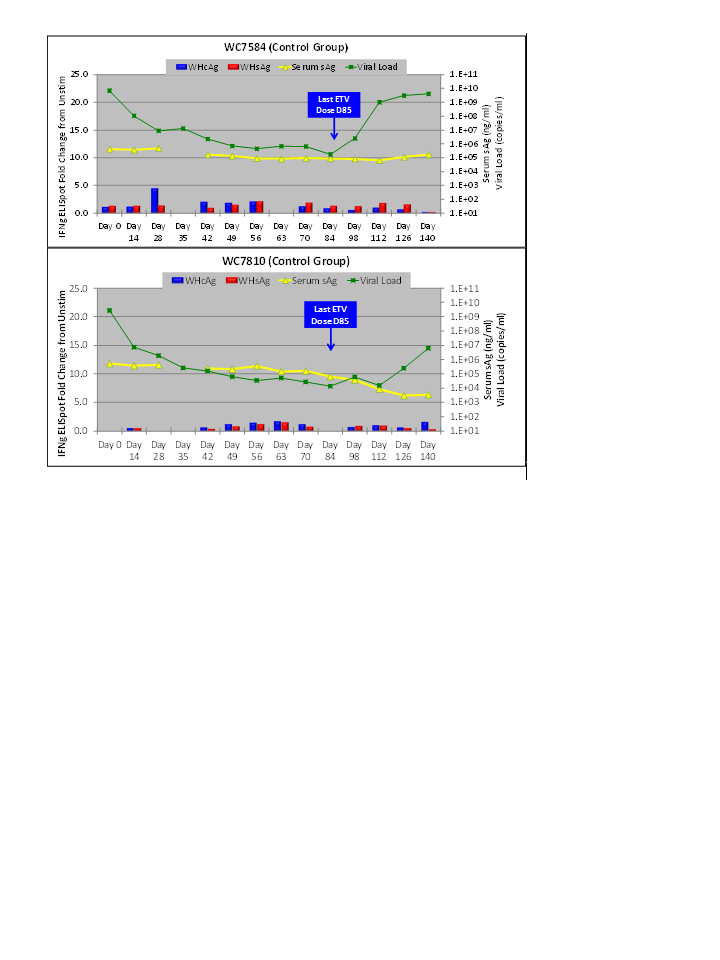

Supplement: S7 Fig — PBMCs were isolated and tested for responses to WHV core and sAg peptide libraries as described in Materials and Methods. Results for each individual woodchuck are shown. ELISPOT responses are displayed as fold change over unstimulated controls for WHV cAg library (blue bars) and sAg library (red bars). Viral load (green) and sAg (yellow) in the same animal is overlaid on each graph. Antibody isotype-control treated animals 7584 and 7810. (TIF) [file pone.0190058.s007.tif]

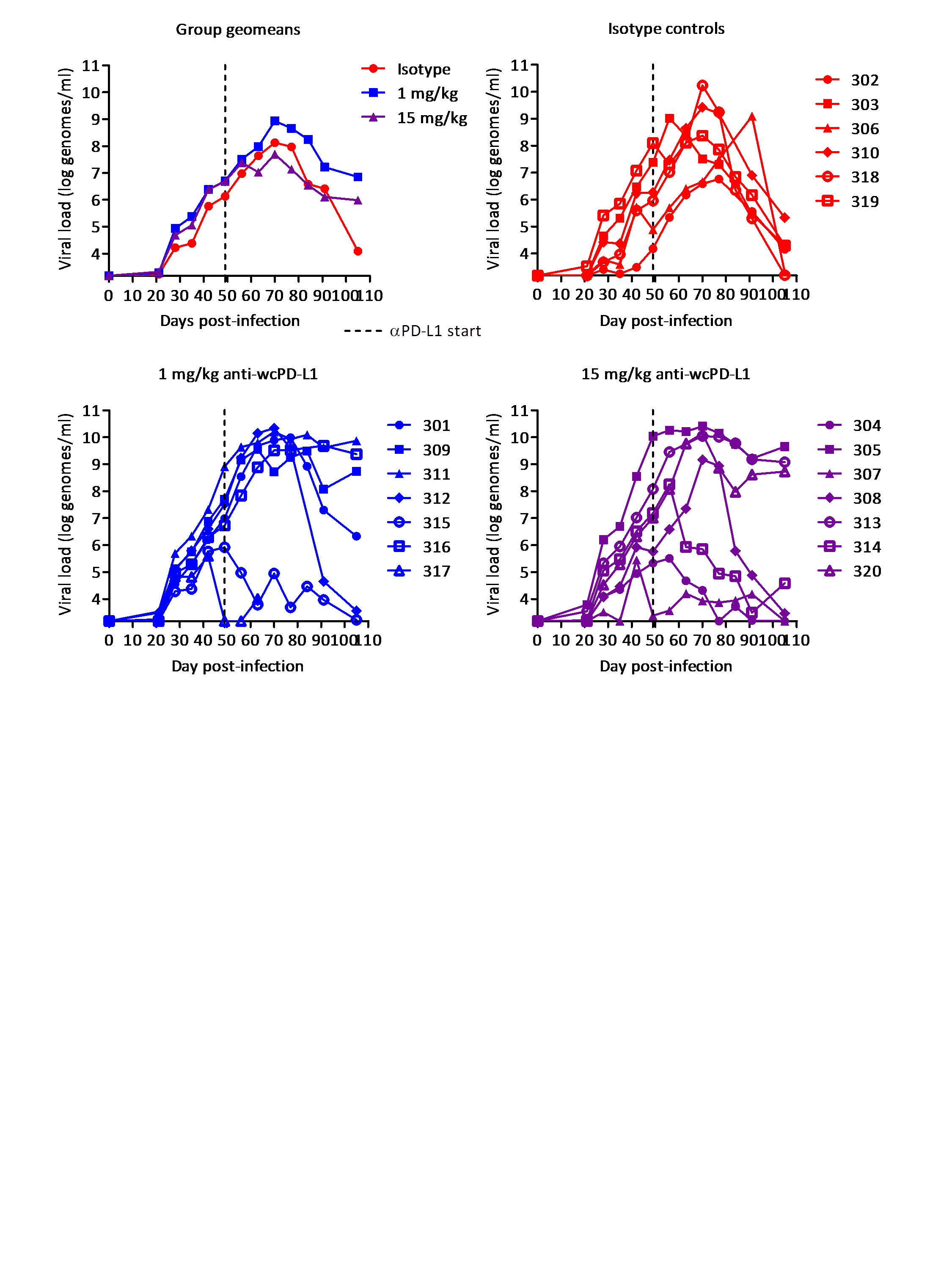

Supplement: S8 Fig — Animals undergoing acute WHV infection were treated with wc6D5 at 1 mg/kg (blue) or 15 mg/kg (purple), or with isotype control MAb wc6D5 at 15 mg/kg (red). Antibodies were administered in four doses over 10 days, starting on week 7 post-WHV infection. Viral loads for each individual animal in each group are shown. (TIF) [file pone.0190058.s008.TIF]

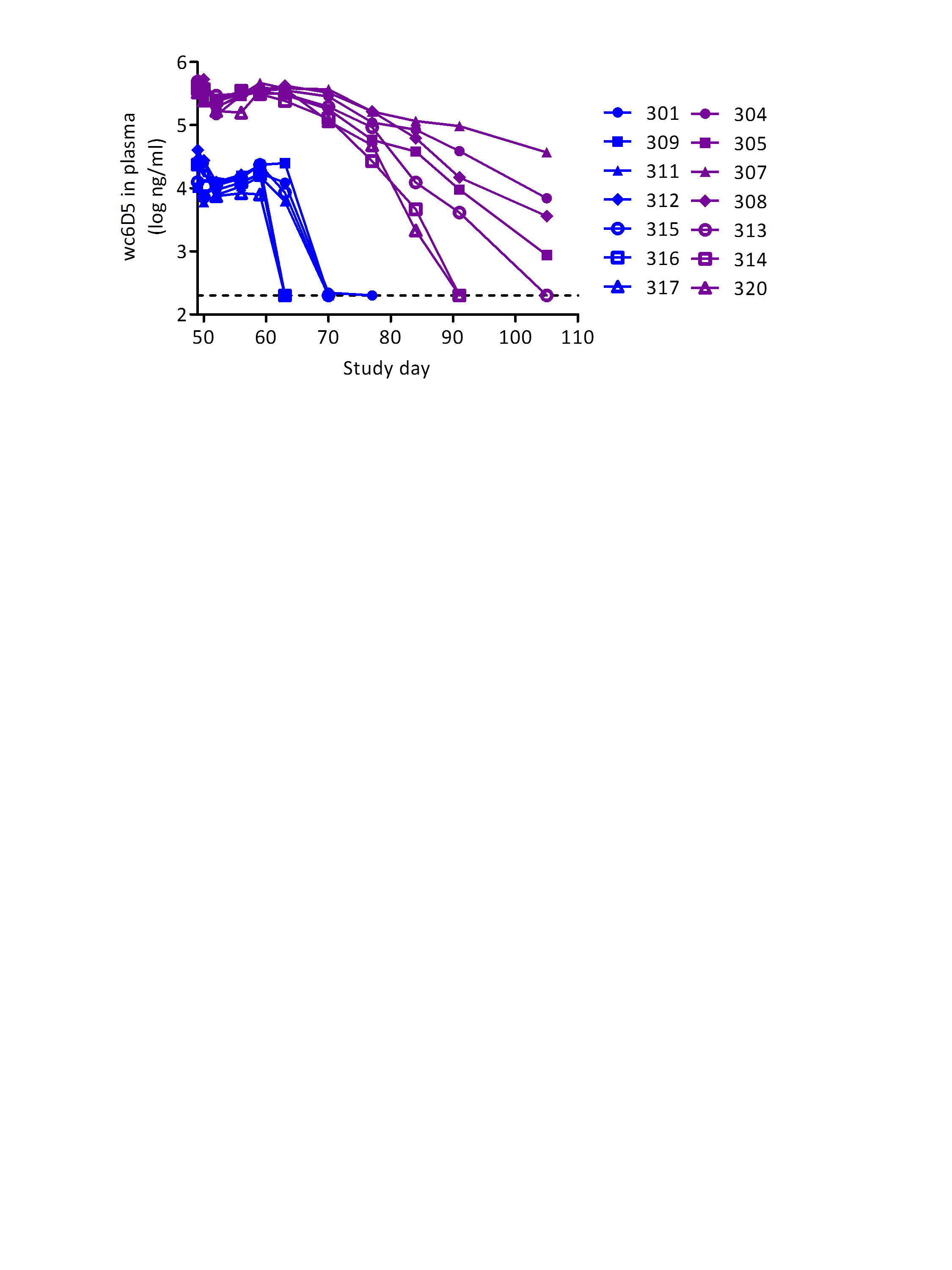

Supplement: S9 Fig — Plasma levels of anti-woodchuck PD-L1 mAb wc6D5 was determined in plasma of treated animals at various times post-infection. Animals received either 1 mg/kg (blue) or 15 mg/kg (purple) wc6D5 in four doses between days 49 and 59 post infection. (TIF) [file pone.0190058.s009.TIF]
